# Explorative study of the sensitivity and specificity of the pronator quadratus fat pad sign as a predictor of subtle wrist fractures

**DOI:** 10.1007/s00256-012-1451-0

**Published:** 2012-06-09

**Authors:** F. Fallahi, H. Jafari, Gail Jefferson, P. Jennings, R. Read

**Affiliations:** 1 Department of Radiology, North Cumbria University Hospitals NHS Trust, Carlisle, UK; 2 Emergency Department, North Cumbria University Hospitals NHS Trust, Carlisle, UK; 3 University of Cumbria, Carlisle, UK; 4 Department of Radiology, The Cumberland Infirmary, Newtown Road, Carlisle, Cumbria CA2 7HY UK

**Keywords:** Pronator quadratus muscle, Pronator quadratus fat pad, Wrist fracture, MRI of the wrist

## Abstract

**Objective:**

To investigate the value of post-traumatic pronator quadratus (PQ) fat pad sign as a reliable predictor of subtle wrist fractures.

**Materials and methods:**

This was a prospective study of 68 patients undergoing X-ray for traumatic wrist injuries and subsequent MRI. The reliability of a positive PQ fat pad sign on X-ray, defined as either raised, interrupted or obliterated, was evaluated in detection of subtle wrist fractures.

**Results:**

Out of 68 patients, 28 had a positive PQ sign without any obvious bony injuries on plain radiographs; of these, the PQ fat pad was obliterated in 11, disrupted in 12, and raised in five cases. Fractures defined as cortical interruption or trabecular fractures were revealed in 13/28 (46 %) patients with a positive PQ sign but only in 7/40 (18 %) patients with a negative sign. With regards to different types of abnormal PQ fat planes, fractures were present in 7/12 patients with a disrupted plane (58 %), 6/11 patients with an obliterated plane (54 %), and none of the patients with a raised plane. The overall sensitivity and specificity of a positive PQ sign in the prediction of occult wrist fractures were 65 and 69 %, respectively.

**Conclusions:**

Our findings indicate that a positive pronator quadratus (PQ) fat pad sign is not a reliable predictor of subtle fractures of the wrist, although we believe that it is a useful radiographic sign in justifying MRI for further clarification of possible joint abnormalities including occult fracture and cortex interruption.

## Introduction

**Fig. 1:**
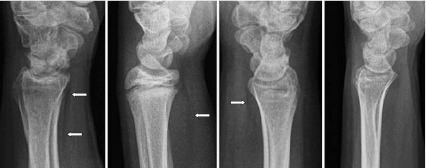
From* left to right*: normal, raised, disrupted, and obliterated PQ fat planes

The pronator fat pad is a small fat plane at the volar aspect of the distal forearm. A fracture of the distal forearm can displace this and create a radiologically “positive” fat pad sign (Fig. 1).

The pronator quadratus (PQ) fat pad (FP) sign was first described in 1963 by MacEwan who observed an altered FP sign in 295 of 300 patients with fractures at the level of PQ attachment [1]. Figure 1 shows lateral radiographs of the wrist with a normal, raised, disrupted, and obliterated PQ fat plane. In 1984, Curtis et al. evaluated the importance of soft-tissue changes in wrist trauma and reported a positive PQ sign in 80 % of distal forearm fractures [2]. Also in 1984, Zimmer studied fat plane radiological signs in wrist and elbow trauma and addressed conditions that could create false-positive and false-negative FP signs [3]. A false-positive fat pad sign can be due to arthritides, local infections, localized inflammations and severe soft-tissue injuries. Fractures not lying under the PQ muscle, poor-quality radiographs, and radiographs taken immediately after injury can produce a false-negative PQ sign.

Zammit-Maempell et al. [4] measured the pronator value (maximum distance between the pronator fat line and the distal radius) in 1,453 patients with wrist injury and reported a statistically significant increased pronator value in fractures of the distal forearm.

In their study, the pronator FP was abnormal in only 51 % of forearm fractures. Sasaki and Sugioka [5] classified the radiological appearances of the PQ sign into four types ranging from an undisplaced straight or gently convex curve to an absent soft-tissue shadow.

To our knowledge, only one study has been published on the reliability of PQ sign [6]. This study used MRI of the wrist to confirm occult fractures. Plain radiographs of 50 patients with a proven fracture confirmed by MRI were examined for the presence of a PQ sign. The PQ sign was positive in 20 %, absent in 6 %, and negative in 74 %. The pronator FP was abnormal in 15 (30 %) of another 50 patients whose MRI showed no fracture. Sensitivity and specificity of this sign were calculated as 26 and 70 %, respectively. The authors concluded that the PQ sign was a poor predictor of underlying occult distal radial fracture.

Our study was aimed to assess the reliability of the PQ fat pad sign in detecting subtle fractures of wrist, which are not visible on an initial a radiograph, but can, however, be confirmed by MRI examination.

## Methods

### Study design

A controlled prospective observational study was conducted in the Emergency Department (ED) and Department of Radiology of Cumberland Infirmary in Carlisle (CIC), UK, between July 2008 and July 2010. Approval was obtained from the National Research Ethics Committee, reference number 08/H1015/20. A patient information sheet was provided to each patient with exact explanation of the purpose of the study, clear message of voluntary principal of participation, and careful discussion of contraindications of MRI. Informed written consent was obtained from all patients or their legal guardians.

### Study setting and population

CIC is a teaching hospital with an ED census of 41,000 visits per year. The hospital is the main part of the North Cumbria University Hospitals serving a population of 330,000. Patients presenting to the ED with wrist injuries whose plain radiographs showed an altered PQ fat plane and no obvious bony fracture were included. We classified the PQ fat plane abnormalities into one of three categories: raised, disrupted, and obliterated. All these pathological alterations are due to soft-tissue swelling with or without bleeding from a fracture site resulting in a positive PQ sign. The shape and form of different types of PQ fat plane can be appreciated on Fig. 1. If more than one characteristic was present, then for analysis purposes, an obliterated fat plane overrode disrupted and raised planes, and a disrupted plane overrode a raised plane. A preliminary study of radiographs of hand and wrist of 60 patients (30 males, and 30 females) without any preceding trauma, commonly showed PQ values up to 8 mm in females and 9 mm in males, therefore in our study, a PQ fat plane value greater than 8 mm in females and greater than 9 mm in males considered as raised. Patients with active osteoarthritis, inflammatory arthritis, osteomyelitis of the wrist, and children younger than 16 years of age were excluded.

### Study protocol

At CIC, all trauma radiographs are reported within 24 h and a copy of the report is sent to the ED so that any patients with missed injuries are reviewed immediately and receive the appropriate management. Radiographs of patients with wrist injury and an altered PQ fat plane but without evidence of any obvious fracture were selected by a reporting radiographer (GJ) or a consultant radiologist (FF). Standard radiographs in AP and lateral projection were obtained from Philips Optimus Bucky Diagnost or MPS 50 GE Bucky Unit. The average time from injury to X-ray was 1.2 days. Selected patients were then contacted by phone or letter to attend the Department of Radiology for magnetic resonance imaging (MRI) of the injured wrist as soon as possible, and usually within a few days. The control group consisted of patients who had MRI of their wrists as part of investigations for suspected wrist fractures and their initial plain radiographs had revealed a negative PQ sign. Overall, 68 patients were included, 36 males and 32 females, respectively.

MR images were obtained on a 1.5-Tesla Magnetom Avanto Siemens MRI scanner with an 8-channel wrist coil. A standard protocol with coronal T1, coronal PDFS, coronal STIR, sagittal PDFS, and axial PDFS sequences was performed. Magnetic resonance images were reported by two consultant radiologists with a special interest in musculoskeletal radiology (FF and PJ). If the MRI series revealed a significant injury, then the patients were immediately referred to the Fracture Clinic for further management.

## Results

**Fig. 2:**
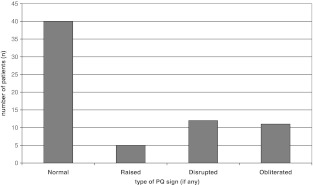
Characteristics of PQ fat signs

Sixty-eight patients were included in our study. Of these, 28 had a positive PQ sign without any obvious bony injuries on plain radiographs. In the group with an abnormal PQ fat pad sign, the fat pad was obliterated in 11, disrupted in 12, and raised in five. In the control group with 40 patients, the PQ sign was negative. The age of patients ranged from 16 to 74, with a mean of 36.5 years. The majority of injuries were sustained following a fall (58 patients), others involved direct blows, twisting and unspecified mechanisms during sporting activities. All plain radiographs were obtained immediately after presentation to the ED and most patients had their MRI within 10 days (53 of 68 patients, 78 %). Only three patients had suffered wrist injuries in the past and two patients had been diagnosed with mild arthritis of the wrist (first carpometacarpal joint in both cases). The characteristics of the PQ fat pad sign on plain radiographs are summarized in Fig. 2.

In the group with a positive PQ sign, MRI was normal in five patients, revealed only joint effusion in four patients, bone bruising in five, cortical interruption in seven, trabecular fracture in six, and scapholunate ligament tear in one. In the group with a negative PQ sign, MRI was normal in 23 patients, revealed bone bruising in five, trabecular fracture in five, cortical interruption in two, radial collateral ligament tear in three, scapholunate ligament tear in one, and synovitis in one. For the purpose of the study, MRI was considered normal when only degenerative changes had been shown. Table 1 demonstrates the frequency of different MRI findings with different PQ signs.

**Table 1:** Frequency of MRI findings of wrist images

	Negative PQ sign (*n*/%)		Positive PQ sign (*n*/%)	
Normal	23	57.5 %	5	17.8 %
Bone bruise	5	12.5 %	5	17.9 %
Trabecular fracture (#)	5	12.5 %	6	21.4 %
Radial collateral ligament tear	3	7.5 %	0	0 %
Cortical interruption (#)	2	5.0 %	7	25 %
Synovitis	1	2.5 %	0	0 %
Scapholunate ligament tear	1	2.5 %	1	3.6 %
Joint effusion	0	0 %	4	14.3 %
Total	40		28	

Bone bruising, cortical interruption, and trabecular fractures were noted in distal radius or ulna, scaphoid, lunate, hamate, or a combination of the above sites. The pronator value as described by Zammit-Maempell et al. [4] ranged from 8 to 11.5 mm in 17/28 cases. This value was higher in injuries with bone bruising or occult fracture compared to soft-tissue injury or joint effusion. Fractures defined as cortical interruption or trabecular fractures were revealed in 13/28 (46 %) patients with a positive PQ sign and in 7/40 (18 %) patients with a negative sign. With regards to different types of abnormal PQ fat planes, fractures were present in 7/12 patients with a disrupted plane (58 %), 6/11 patients with an obliterated plane (54 %) and none of the patients with a raised plane.

The sensitivity and specificity of a positive PQ sign in predicting occult fractures of wrist were 65 and 69 %, respectively. These values were calculated for obliterated, disrupted and raised pronator quadratus fat planes as individual markers for a positive PQ sign as shown in Table 2.

**Table 2:** Sensitivity and specificity of a positive PQ sign in predicting subtle fractures of wrist in each subgroup and in total

PQ fat pad sign	Sensitivity	Specificity
Obliterated PQ	30 %	89 %
Disrupted PQ	35 %	89 %
Raised PQ	0 %	89 %
Any positive PQ sign	65 %	69 %

## Discussion

A positive pronator quadratus (PQ) fat pad sign does not seem to be a reliable predictor of a subtle fracture of the wrist. When subgroups of this sign are taken into consideration individually, then all of them are sufficiently specific signs to suggest subtle fractures of the wrist. A considerable number of the patients (13/23) in the subgroups of obliterated and disrupted PQ fat pad sign showed significant bone injuries, which required immediate referral to the Fracture Clinic for further management. A higher specificity of all three subgroups (89 %) justifies performing MR imaging of the wrist to further investigate the possibility of a subtle fracture such as cortical interruption or trabecular fracture. The individual subgroups still suffer a low sensitivity and therefore a negative fat pad sign should not be used to rule out the possibility of a fracture.

A major limitation of our study was the fact that a sample size was not calculated to give a sufficient power to the study; more patients would be needed to achieve statistical significance.

Although a positive PQ sign in many cases occurs due to bone bruising, joint effusion or soft-tissue injuries, the majority of PQ sign positive patients in our study had significant bone injuries. Therefore we propose that in the presence of an abnormal PQ fat pad sign, MRI of the wrist is justified to detect occult fractures and to ensure high-quality care.

## Conclusions

Our findings demonstrate that a positive pronator quadratus (PQ) fat pad sign is not a reliable predictor of subtle fracture of the wrist. However, we believe that it is a useful radiographic sign and justifies MRI for further evaluation of intraarticular abnormalities including occult fracture and cortex interruption, to ensure optimum patient management.
